# Generating in vitro models of NTRK-fusion mesenchymal neoplasia as tools for investigating kinase oncogenic activation and response to targeted therapy

**DOI:** 10.1038/s41389-023-00454-6

**Published:** 2023-02-17

**Authors:** Fabio Vanoli, Laurie Herviou, Yusuke Tsuda, Patricia Sung, Ziyu Xie, Eve Fishinevich, Soe S. Min, William Mallen, Henry de Traux de Wardin, Yanming Zhang, Maria Jasin, Cristina R. Antonescu

**Affiliations:** 1 Department of Pathology, Memorial Sloan Kettering Cancer Center, New York, NY USA; 2 Developmental Biology Program, Sloan Kettering Institute, New York, NY USA

**Keywords:** Molecular biology, Cancer, Stem cells, Sarcoma

## Abstract

The discovery of neurotrophic tyrosine receptor kinase (NTRK) gene fusions as pan-tumor oncogenic drivers has led to new personalized therapies in oncology. Recent studies investigating *NTRK* fusions among mesenchymal neoplasms have identified several emerging soft tissue tumor entities displaying various phenotypes and clinical behaviors. Among them, tumors resembling lipofibromatosis or malignant peripheral nerve sheath tumors often harbor intra-chromosomal *NTRK1* rearrangements, while most infantile fibrosarcomas are characterized by canonical *ETV6::NTRK3* fusions. However, appropriate cellular models to investigate mechanisms of how kinase oncogenic activation through gene fusions drives such a wide spectrum of morphology and malignancy are lacking. Progress in genome editing has facilitated the efficient generation of chromosomal translocations in isogenic cell lines. In this study we employ various strategies to model *NTRK* fusions, including *LMNA::NTRK1* (interstitial deletion) and *ETV6::NTRK3* (reciprocal translocation) in human embryonic stem (hES) cells and mesenchymal progenitors (hES-MP). Here, we undertake various methods to model non-reciprocal, intrachromosomal deletions/translocations by induction of DNA double strand breaks (DSBs) exploiting either the repair mechanisms of homology directed repair (HDR) or non-homologous end joining (NHEJ). Expression of *LMNA::NTRK1* or *ETV6::NTRK3* fusions in either hES cells or hES-MP did not affect cell proliferation. However, the level of mRNA expression of the fusion transcripts was significantly upregulated in hES-MP, and phosphorylation of the *LMNA::NTRK1* fusion oncoprotein was noted only in hES-MP but not in hES cells. Similarly, an *NTRK1*-driven transcriptional profile related to neuronal and neuroectodermal lineage was upregulated mainly in hES-MP, supporting the importance of appropriate cellular context in modeling cancer relevant aberrations. As proof of concept of the validity of our in vitro models, phosphorylation was depleted by two TRK inhibitors, Entrectinib and Larotrectinib, currently used as targeted therapy for tumors with *NTRK* fusions.

## Introduction

*NTRK* gene fusions have been identified in a diverse range of pediatric and adult tumor types, including benign and malignant mesenchymal neoplasms of either fibroblastic or neural line of differentiation [1–5]. These fusions result from inter- or intra-chromosomal rearrangements leading to juxtaposition of the 3’ region of an *NTRK* gene (encoding the full kinase domain) with the 5′ region of a partner gene (encoding an oligomerization or other protein-association domain), ultimately producing a constitutively active TRK fusion protein that activate the PI3K and the MAPK pathways. Moreover, some studies have shown that *NTRK* genotype may correlate with tumor phenotype and risk of malignancy, with *NTRK1* intrachromosomal rearrangements (interstitial deletions or inversions) occurring in most benign lipofibromatosis-like neural tumors [3], while *ETV6::NTRK3* fusions driving overwhelmingly malignant lesions [2]. Despite the heterogeneity in phenotypes and clinical behaviors, most mesenchymal tumors driven by *NTRK* fusions lack other secondary genetic alterations. However, due to the lack of faithful experimental models, the mechanisms of how *NTRK*-oncogenic activation through various intra- or inter-chromosomal translocations result in tumors of various cell lineages and outcomes remain elusive. In this study we generate isogenic cell lines driving the oncogenic activation of TRK kinases, which are responsible for infantile fibrosarcoma and other emerging novel spindle cell tumors with *NTRK* fusions [3].

## Results

### Generation of the *LMNA::NTRK1* fusion in hES cell lines using a conditional expression system

**Fig. 1:**
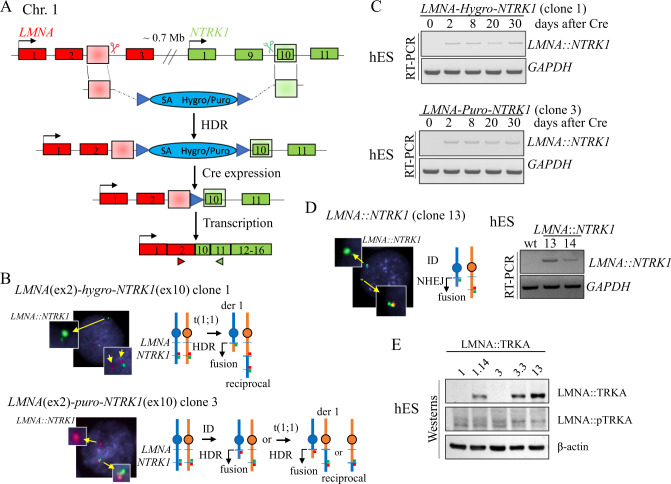
Generation of hES cell lines with conditional *LMNA::NTRK1* fusion expression. **A** Strategy for conditional *LMNA::NTRK1* fusion gene expression. A donor template with homology arms (shaded boxes) and a promoter-less selectable marker (hygromycin or puromycin) is introduced at the DSB sites (scissors) by HDR. A splice acceptor (SA) sequence allows the expression of the marker from the *LMNA* promoter. Induction of the fusion occurs after removal of the selectable marker by expression of the CRE recombinase. **B** Dual color FISH analysis showing the *NTRK1* locus and the rearranged allele in clones 1 (hygromycin positive; red, centromeric; green, telomeric) and 3 (puromycin positive; green, centromeric; red, telomeric) after loss of the centromeric probe either by t(1;1) or ID. **C** RT-PCR analysis in hES cells showing the *LMNA::NTRK1* fusion level over a period of 30 days after expression of the CRE recombinase in clones 1 and 3. **D** Dual color FISH analysis on clone 13, (from NHEJ strategy, see S1D) showing loss of 5′ *NTRK1* probe (red, centromeric) and RT-PCR confirming the presence of the *LMNA::NTRK1* fusion in two distinct clones. **E** Western blot analysis showing the LMNA::TRKA fusion protein in clones isolated after removal of the marker (clones 1.14 and 3.3) and clone 13. Phosphorylation of TRKA is not detected.

*LMNA::NTRK1* fusion is the most common genetic event driving mesenchymal tumors with a lipofibromatosis-like neural phenotype [3, 6]. The *LMNA* and *NTRK1* genes are located on 1q21.2 locus (0.7 Mb apart) with the same direction of transcription. Based on prior targeted RNA/DNA sequencing and FISH studies, it was suggested that the leading mechanism of *LMNA::NTRK1* fusion in sarcomas is through a 0.7 Mb interstitial deletion (ID) [6, 7], rather than a reciprocal t(1;1) translocation. We first applied a similar strategy designed for selection of reciprocal inter-chromosomal translocations [8]. Briefly, a donor template with a splice acceptor (SA) site was used resulting in the expression of a selectable marker (hygromycin or puromycin) from the *LMNA* promoter upon correct integration (Fig. 1A). This gene trap strategy allows the expression of the selectable marker while blocking the fusion expression that can be conditionally induced by Cre recombinase-mediated removal of the marker. Integration of the donor template is promoted by induction of double strand breaks (DSBs) in the participating loci. Relevant to the most common fusion transcript reported in human tumors [7, 9–11], we designed sgRNAs to induce DSBs in intron 2–3 of *LMNA* and intron 9-10 of *NTRK1*, adjacent to the sequences homologous to the donor (shaded boxes), to generate the *LMNA* (exon2)-*NTRK1* (exon10) isoform (Fig. 1A). The *LMNA* intron 2–3 and the *NTRK1* intron 9-10 sequences were screened for the presence of interspersed repeats and low complexity DNA sequences that could impair targeting efficiency (www.repeatmasker.org), and the sgRNAs were selected using the Guidescan website [12].

### Generating *LMNA::NTRK1* fusions in hES cells at improved efficiency

Selection of hygromycin positive clones proved to be inefficient compared to prior targeting experiments modeling other sarcoma-related gene fusions in hES cells [13], with <10 colonies/nucleofection of irregular shape and reduced plating efficiency after the first passage. Out of the surviving colonies only one clone (clone 1) showed correct integration of the donor template by PCR analysis. Fluorescence In Situ Hybridization (FISH) assays using custom BAC probes flanking both *NTRK1* and *LMNA* genes (centromeric, red probe; telomeric, green probe) showed a signal pattern consistent with a t(1;1) translocation rather than an ID (Figs. 1B and S1A). The presence of the reciprocal chromosome was confirmed by PCR and sequencing of the breakpoint junction between *LMNA* intron 2–3 and *NTRK1* intron 9–10 (Fig. S1B).

The donor template was modified to include a puromycin selectable marker and a longer *LMNA* homology arm (461 bp instead of 372 bp). After 10-day selection, 175 colonies were recovered, with 11 clones showing correct integration of the donor template at both participating loci. The puromycin resistant colonies revealed a uniform, rounded shape, and grew after several passages. One clone (clone 3) analyzed by FISH showed an unbalanced rearrangement in keeping with an ID. However, it is possible that the event occurred through a translocation involving multiple DSBs, leading to the imbalance (Fig. 1B). Moreover, the junction of the reciprocal translocation could not be detected, in contrast to the t(1;1) translocation-associated clone 1 (Fig. S1B).

### *LMNA::NTRK1* fusion resulting from either t(1;1) translocation or interstitial deletion (ID) does not affect hES cell proliferation and viability

In contrast to what was observed in hES cells expressing *EWSR1-CREB* fusions [13], *LMNA::NTRK1* transcript levels in both clones (clones 1 and 3) remained stable over a 30-day time course in a pool of cells after Cre recombinase (Fig. 1C). This result suggests that the *LMNA::NTRK1* fusion does not impair cell proliferation and/or viability of hES cells, unlike *EWSR1-CREB* fusions. We then isolated clones that constitutively express the *LMNA::NTRK1* fusion after removal of the selectable marker by Cre recombinase. Two clones from each cell line were tested showing comparable *LMNA::NTRK1* fusion transcript levels by RT-PCR and qRT-PCR (Fig. S1C). One clone from each original cell line (1.14 from clone 1 and 3.3 from clone 3) was selected for further characterization.

### *LMNA::NTRK1* fusions by nonhomologous end joining (NHEJ) repair of DSBs

Given the viability of cells expressing the fusion, we also attempted to directly model the fusion by generating two DSBs in the intronic regions of *LMNA* and *NTRK1* in the absence of a homologous donor to allow repair of DNA ends by NHEJ (Fig. S1D). PCR screening by breakpoint junction amplification confirmed the presence of deletions in two clones from a 96-well plate (Fig. S1D). FISH analysis showed the same pattern as observed for clone 3, with loss of the centromeric portion of the *NTRK1* gene, and RT-PCR showed similar expression of the *LMNA::NTRK1* fusion in both clones (Fig. 1D). One clone (clone 13) was selected for comparison with clones 1.14 and 3.3 from the previous targeting strategies.

### Induction of the *LMNA::NTRK1* fusion in Retinal Pigment Epithelium (RPE) cell line

As the *LMNA::NTRK1* fusion has been identified in different tumor types including carcinoma, we modeled the fusion in epithelial cells and evaluated the effect on cell proliferation/viability. Retinal Pigment Epithelium (RPE) cells were transfected with sgRNA for *LMNA* and *NTRK1* and the fusion transcript resulting from NHEJ repair was monitored in a time course experiment in a pool of transfected cells. The fusion product was detected up to 18 days, before reduction at day 21 and disappearance by day 25 (Fig. S1E). We also attempted single-cell plating from transfected cells to select single clones expressing the fusion but none of the 112 clones analyzed by PCR showed amplification at the breakpoint junction. These results suggest that the *LMNA::NTRK1* fusion affects differently the cell proliferation and/or viability depending on the cellular background.

### Detection of the LMNA::TRKA fusion protein in hES clones

Western blot analysis using an antibody against the C-terminal, catalytic portion of TRKA protein confirmed the presence of the oncogenic fusion protein in clones expressing the fusion transcript (clones 1.14, 3.3 and 13), but not in the parental clones not treated with Cre recombinase (clones 1 and 3) (Fig. 1E). The TRKA kinase domain encompasses exon 12 to 15 with exon 13 coding for the ATP-binding pocket and exon 14 for the activation loop. While we confirmed by Sanger sequencing that the exons are included in the fusion transcripts of the clones 1.14, 3.3 and 13 (Fig. S2), western blot analysis using phospho-TRKA antibodies shows that the TRKA residues Tyr680/681, typically activated in fusion positive tumors, were not phosphorylated in hES cells (Fig. 1E).

### *LMNA::NTRK1* fusion expression and protein localization in hES-cell derived mesenchymal progenitors

**Fig. 2:**
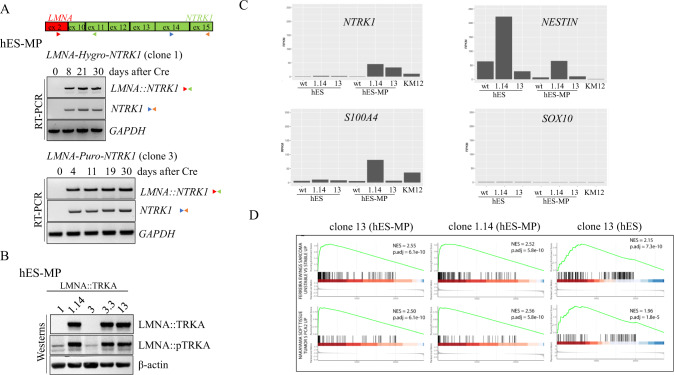
Expression of *LMNA::NTRK1* fusion in human mesenchymal progenitors (hES-MP). **A** Diagram of *LMNA::NTRK1* transcript with primers position for RT-PCR. Time course (days) experiment for *LMNA::NTRK1* fusion and *NTRK1* expression in hES-MP (clones 1 and 3) after Cre expression. **B** Western blot showing LMNA::TRKA product and phosphorylation of TRKA in hES-MP cells expressing the fusion transcript. **C** Histogram plot from RNAseq analysis showing *NTRK1, NESTIN, S100A* and *SOX10* RNA levels in hES and hES-MP expressing the *LMNA::NTRK1* fusion compared to wild-type isogenic cell line and colorectal cancer cell line KM12 expressing the *TPM3::NTRK1* fusion. **D** GSEA analysis for cell lines expressing the *LMNA::NTRK1* fusion (clones 13 and 1.14 (hES-MP) and 13 (hES)). GSEA shows an enrichment of pathways involved in Ewing sarcoma and soft tissue sarcoma.

The use of hES-MP to model cancer relevant translocations and to study the role of fusions in oncogenesis is hampered by their limited passages. We surmounted these limitations by using hES cells that can grow almost indefinitely, form colonies from single cells and differentiate into multiple cell lines. To functionally characterize the role of the *LMNA::NTRK1* fusion in tumor development, hES cell models were differentiated to mesenchymal progenitors (hES-MP), the putative cell of origin of different mesenchymal tumor entities expressing this fusion. Cre recombinase was expressed in hES-MP by either transfection or infection, and fusion transcript level was monitored for 30 days. As with hES cells, the *LMNA::NTRK1* transcript levels were constant over time in hES-MP cells, without perturbation of cell proliferation and with the *NTRK1* portion expressed from the fusion transcript and not from the endogenous allele (Fig. 2A). However, distinct from what was observed in hES cells, phosphorylation of TRKA was detected in these differentiated mesenchymal progenitors (Fig. 2B), emphasizing the importance of the appropriate cellular background when modeling cancer relevant alterations. Immunohistochemistry experiments demonstrated that the cytoplasmic/perinuclear positivity of the LMNA::TRKA fusion protein in a variety of tumor types, including carcinoma and sarcoma, is an effective diagnostic tool [14, 15]. The immunohistochemistry with pan-TRK antibodies on hES-MP cells expressing the *LMNA::NTRK1* fusion (clone 3.3) confirmed the cytoplasmic/perinuclear localization of the fusion protein (Fig. S3A).

### *LMNA::NTRK1*-expression in hES-MP cells drives an *NTRK1*-transcriptional profile with upregulation of neuronal and neuroectodermal signatures

Whole transcriptome sequencing (RNAseq) was performed on clones 1.14 and 13 in both the hES and hES-MP backgrounds. Individual comparisons between the *LMNA::NTRK1* positive clones versus wild-type hES and hES-MP cell lines and KM12 colorectal cancer cell line expressing the *TPM3::NTRK1* fusion demonstrated differentially expressed genes (DEG) using log2 fold change (FC) > 2 (Fig. 2C). For example, *NTRK1* mRNA, presumably from the fusion transcript, was highly upregulated in *LMNA::NTRK1* hES-MP compared to wild-type hES-MP cells or to the hES background (Fig. 2C), which was confirmed by RT-PCR for the *LMNA::NTRK1* fusion in clones 1.14, 3.3, and 13 (Fig. S3B). Similar to the consistent immunoprofile of *LMNA::NTRK1* driven human mesenchymal tumors [3, 6, 16], clone 1.14, although not clone 13, showed mRNA upregulation of S100 but no expression of SOX10 (Fig. 2C). Moreover, *NESTIN* mRNA levels were upregulated in clones 1.14 and less so in clone 13 compared to wild-type hES-MP cells (Fig. 2C), similar to what has been previously reported in *NTRK*-fusion positive tumors [7].

The individual DEG and their log2FC from each comparison described above were then subjected to gene set enrichment analysis (GSEA) using Bioconductor cluster Profiler to identify significant pathways. The upregulated genes in hES-MP cells expressing the *LMNA::NTRK1* fusion (clone 13 and 1.14) were found to be enriched among the statistically significant pathways involved in neuronal development, neural crest stem cells, Ewing sarcomas and other soft tissue sarcomas (Fig. 2D and Supplementary Table S1).

### Modeling the canonical *ETV6::NTRK3* gene fusion in hES and hES-MP cells

**Fig. 3:**
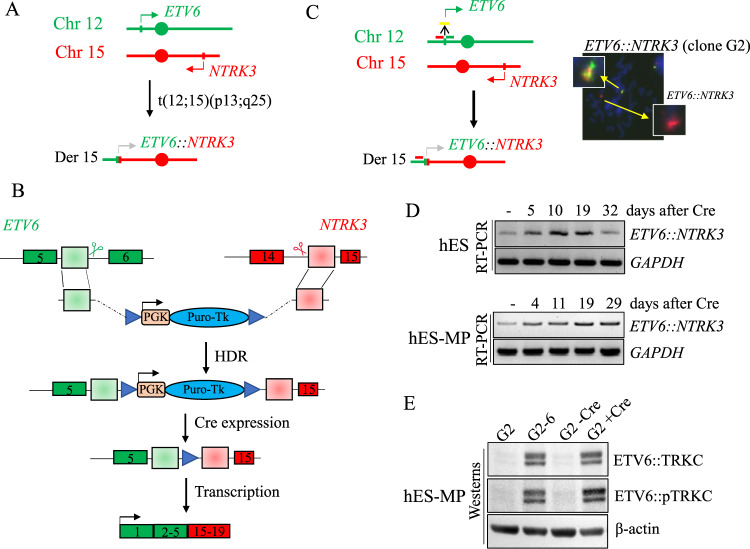
Generation of the *ETV6::NTRK3* chromosomal translocation in hES cells and differentiation to hES-MP. **A** Breakpoints within *ETV6* on chromosome 12 and *NTRK3* on chromosome 15 create the *ETV6::NTRK3* t(12;15)(p13;q25). **B** A donor template containing a puromycin selectable marker under the control of a PGK promoter is inserted by HDR at the *ETV6* and *NTRK3* participating loci after induction of DSBs by sgRNA (scissors). The selectable marker is removed by Cre recombinase allowing the expression of the oncogenic fusion. **C** Dual color FISH analysis with telomeric (red) and centromeric (green) probes on *ETV6* showing the *ETV6::NTRK3* translocation on clone G2. **D** RT-PCR time course (days) for *ETV6::NTRK3* fusion after Cre expression in hES and hES-MP cells. The fusion product is detectable in cells before Cre recombination indicating a partial splicing of PGK-Puro sequence. **E** Western blot analysis showing the ETV6::TRKC fusion protein and the TRKC phosphorylation in hES-MP clone G2-6 and G2 after expression of Cre recombinase. The total ETV6::TRKC protein was detected by pan-TRK antibodies and the TRKC phosphorylation by phospho TRKA/B antibodies (same used for detection of LMNA::pTRKA).

The *ETV6::NTRK3* fusion represents the canonical fusion (90% frequency) for both infantile fibrosarcoma and mammary analog secretory carcinoma, while being detected at a much lower frequency (5–10%) in a variety of other cancers of various lineages [17]. The *ETV6::NTRK3* fusion results from a balanced t(12;15)(p13;q25) translocation, encompassing the entire kinase domain of TRKC expressed by exons 15–18 of *NTRK3*, with the ATP-binding pocket on exon 16 and the activation loop on exon 17 (Figs. 3A and S2). Employing a similar methodology used to model the desmoplastic small round cell tumor *EWSR1::WT1* fusion [8], we aimed to fuse *ETV6* exons 1–5 to *NTRK3* exons 15-19 in hES cells (Fig. 3B). Briefly, a donor template with homology arms was transfected into hES cells and integrated by HDR at the participating loci. The donor template carries a puromycin selectable marker that is expressed under the control of a PGK promoter and can be excised by Cre recombinase. From the 56 puromycin resistant clones, the PCR analysis showed one clone harboring the translocation (clone G2), which was confirmed by the break-apart FISH analysis (Fig. 3C). The reciprocal translocation (der 12) was not detected in this clone. Cells were then differentiated to hES-MP progenitors, and the fusion transcript levels were monitored in both cell types, remaining stable for a 30-day period (Fig. 3D). Of note, the fusion transcript was detectable in both cell types before Cre expression at consistently lower levels compared to post-Cre cells, likely due to a partial splicing to delete the selectable marker [8]. After Cre expression, hES clones that had removed puromycin marker were isolated (clone G2-6) and differentiated to hES-MP. Immunohistochemistry with pan-TRK antibodies confirmed the nuclear localization of the ETV6::TRKC fusion protein observed in tumor samples [14, 15] (Fig. S3C). Comparison of the fusion transcript levels between the two cellular backgrounds (hES and hES-MP) showed a higher level of *ETV6::NTRK3* fusion in mesenchymal progenitors (Fig. S3D), as for *LMNA::NTRK1*. The induction of the ETV6::TRKC protein and the phosphorylation of the TRKC portion were detected by western blot in hES-MP from clone G2-6 and G2 cells infected with Cre recombinase (Fig. 3E).

### Inhibition of TRK-phosphorylation by pan-TRK inhibitors

**Fig. 4:**
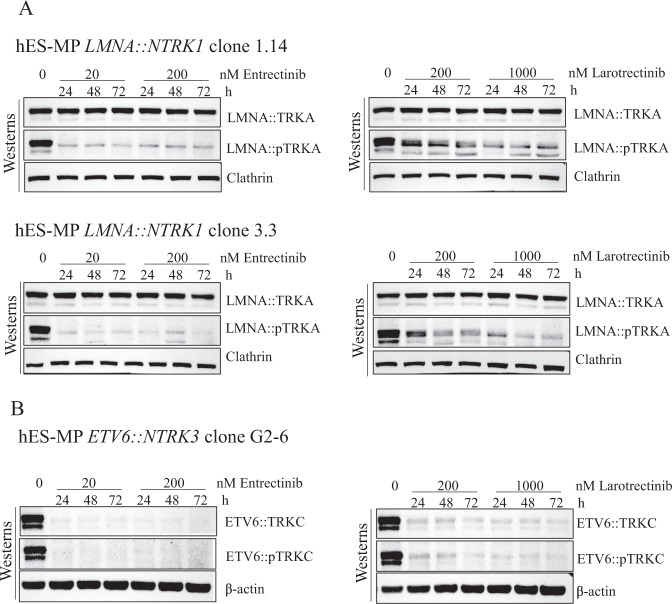
Response to Entrectinib and Larotrectinib inhibition of hES-MP cells expressing LMNA::TRKA and ETV6::TRKC fusion proteins. **A** Western blot analysis showing the LMNA-TRKA fusion protein and the loss of TRKA phosphorylation in hES-MP clones 1.14 and 3.3 after 24, 48, and 72 h treatment with the TRK inhibitors Entrectinib (0, 20, and 200 nM) and Larotrectinib (0, 200, and 1000 nM). **B** Reduced ETV6::TRKC protein stability and TRKC phosphorylation in hES-MP clone G2-6 at different doses of Entrectinib and Larotrectinib.

To confirm that the isogenic hES-MP cells expressing the *LMNA::NTRK1* and *ETV6::NTRK3* fusion are indeed a bona fide model for human tumors driven by the oncogenic activation of the chimeric protein, we monitored TRK phosphorylation in the clones expressing the fusion after treatment with two TRK inhibitors currently used in clinical practice, Entrectinib and Larotrectinib. *LMNA::NTRK1* clone 1.14 was initially exposed to increasing concentrations of both inhibitors. Entrectinib was found to reduce phosphorylation at the lowest dose tested, 5 nM, and Larotrectinib at 100 nM (Fig. S3E). Two concentrations were then chosen for each inhibitor, and cells from two *LMNA::NTRK1* clones (3.3 and 1.14) and the *ETV6::NTRK3* clone G2-6 were treated for 24, 48 and 72 h in a time course experiment. TRKA phosphorylation was reduced in all the conditions tested, while the total fusion protein remained stable (Fig. 4A). Conversely, the stability of ETV6::TRKC was impaired after inhibitor treatment, together with the TRKC phosphorylation [18] (Fig. 4B). To demonstrate the activity of the TRKA-mediated signaling cascade, we tested the phosphorylation of the two well-known downstream effectors ERK1 and ERK2. The phosphorylation of ERK1/2 is constitutively induced in the clone 3.3 expressing the LMNA::TRKA fusion and is reduced to the level of the isogenic clone 3 after a short treatment (1 and 4 h) with Entrectinib and Larotrectinib (Fig. S3F).

**Fig. 5:**
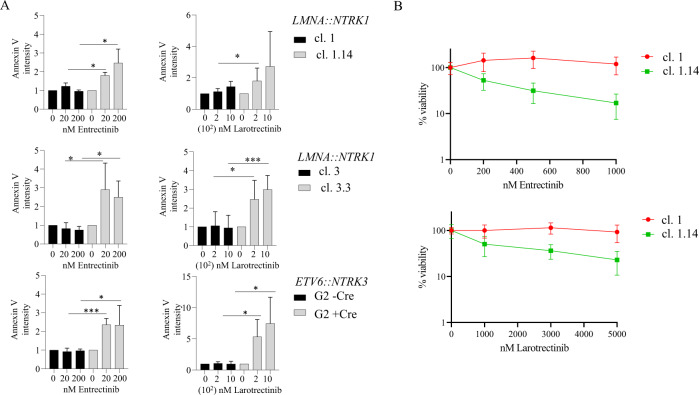
Induction of apoptosis and viability after Entrectinib and Larotrectinib treatment in hES-MP cells expressing LMNA::TRKA and ETV6::TRKC fusion proteins. **A** hES-MP cells from clones 1 and 1.14, 3 and 3.3 and clone G2 before and after expression of Cre recombinase were exposed for 72 h to two doses of Entrectinib (20 and 200 nM) and Larotrectinib (200 and 1000 nM) and Annexin V was quantified as a measure of apoptosis. Annexin V intensity is represented as the ratio of the percentage of untreated cells. Error bars represent standard deviation from the mean of n ≥ 3 independent experiments. Unpaired *t* test *p < 0.05, **p < 0.01, ***p < 0.001, if not indicated the difference is not statistically significant. **B** Cells expressing the LMNA::TRKA protein (clone 1.14) and the corresponding parental cells (clone 1) were treated at increasing concentration of Entrectinib and Larotrectinib for 7 days continuously, before determination of cell viability. Results are presented as the mean of three independent experiments.

We measured apoptosis after 72 h treatment with Entrectinib and Larotrectinib and we found that Annexin V positive cells increased in all cell lines tested (Fig. 5A). Finally, cells expressing the *LMNA::NTRK1* fusion (clones 1.14 and 3.3) were exposed for 7 days continuously to increasing concentration of both inhibitors showing a dose dependent increase in sensitivity when compared to control isogenic cell lines (clone 1 and 3) (Figs. 5B and S3G).

## Discussion

A major breakthrough in the classification of soft tissue tumors has been the recent identification of *NTRK*-fusion related neoplasms which have opened up new avenues for targeted therapy. Although reciprocal translocations resulting in the canonical *ETV6::NTRK3* fusion have been well recognized as the leading driver of infantile fibrosarcoma [19], the emerging group of mesenchymal tumors characterized by intra-chromosomal events at the *NTRK1* 1q23 locus (unbalanced interstitial deletions or cryptic inversions) have been only recently identified as a result of the wide application of RNA sequencing in clinical practice [6]. *LMNA::NTRK1* fusion represents the most common genetic alteration in lipofibromatosis-like neural tumors, a recently described entity with predilection for children and characterized by monomorphic spindle cell phenotype, infiltrating growth pattern, and often co-expression of S100 and CD34 immunomarkers [3, 6]. These tumors are associated with a high local recurrence rate but show a low propensity for distant metastasis [3]. *LMNA::NTRK1* fusion has also been implicated as the driver pathogenetic event in a wide spectrum of tumors of various lineages and risk of malignancies, including carcinomas, melanocytic Spitz lesions, as well as benign and malignant mesenchymal neoplasms [3, 17].

While most oncogenic fusions result from reciprocal chromosomal translocations, unbalanced intrachromosomal interstitial deletions (ID) and even inversions (INV) are also observed. Deletion sizes are quite variable, ranging from submicroscopic and cryptic, being only detectable by next generation sequencing (NGS) (i.e., *STIL::TAL* fusion, 0.019 Mb) [20], to large deletions encompassing numerous genes (i.e., *NDRG1::PLAG1* fusion, 77 Mb) which can be visualized by conventional cytogenetic methods (karyotype, FISH) [21], with the *LMNA::NTRK1* fusion in between (0.7 Mb).

The advent of programmable nucleases (TALENs, Zinc Finger and CRISPR-Cas9) has allowed modeling of cancer-relevant genomic aberrations. For example, the generation of deletions can be achieved by induction of DNA double strand breaks (DSBs), exploiting either the repair by non-homologous end joining (NHEJ) or homology-directed repair (HDR) as shown here. NHEJ represents a fast and reliable method to induce deletions up to ~2 Mb [22], even if laborious sib-selection may be necessary to isolate clones harboring the deletion. NHEJ also relies on viability of cells after fusion formation. In the case of HDR, a donor fragment harboring a selectable marker is inserted, which can prevent fusion protein expression until Cre is expressed. Here we engage both repair mechanisms, given that cells carrying *LMNA::NTRK1* fusions are viable.

Tumors can form from different cell types and the generation of faithful in vitro and in vivo cancer models requires the choice of the correct cellular background. In translocation-associated sarcomas, the cell of origin remains still uncertain with several studies pointing to the mesenchymal compartment [23, 24]. We have recently demonstrated that the use of human embryonic stem mesenchymal progenitors (hES-MP) is important in the generation of Clear Cell Sarcoma (CCS) and Angiomatoid Fibrous Histiocytoma (AFH) models harboring the *EWSR1::ATF1/CREB1* translocations [13]. Using the same approach, we demonstrate that the *LMNA::NTRK1* fusion is expressed at higher level in hES-MP compared to hES cells. Moreover, the phosphorylation of TRKA, a hallmark of tumors expressing the fusion, can be detected only in mesenchymal progenitors. These observations confirm the importance of the cellular context to model cancer relevant aberrations and to study the mechanisms of tumorigenesis.

The *NTRK* genes (*NTRK1-3*) are typically involved in normal neuronal development and encode the tropomyosin receptor kinase (TRK) proteins, a.k.a. TRKA, TRKB, and TRKC, respectively. *LMNA* encodes proteins lamin A and lamin C, which are involved in the nuclear envelope structure [25]. The *LMNA::NTRK1* fusion encodes a coiled-coil dimerization domain of LMNA fused to the tyrosine kinase domain of TRKA. By RNA sequencing, the expression of *LMNA::NTRK1* in the hES-MP background resulted in a transcriptional signature with enrichment of genes involved in neuronal and neural crest function, as well as other upregulated in sarcoma datasets, such as Ewing sarcoma. The latter finding is remarkable, as we have previously demonstrated that Ewing sarcoma shows upregulation of *NTRK1* mRNA and express panTRK at protein level [26]. The enrichment of a neural crest gene signature in the hES-MP expressing the *LMNA::NTRK1* fusion correlates with the neural features displayed by the *LMNA::NTRK1* fusion-positive soft tissue tumors resembling lipofibromatosis-like neural or peripheral nerve sheath tumor phenotypes.

As proof of concept, treatment with TRK inhibitors, including Larotrectinib and Entrectinib, showed a dose-dependent decrease or abrogation of kinase activity of TRKA in hES-MP expressing *LMNA::NTRK1* fusion as well as a reduction of cell viability. The same treatment in hES-MP expressing the *ETV6::NTRK3* fusion leads to ETV6::NTRK3 protein degradation in agreement with previous studies on a role of TRKC in “protecting” the protein from proteosomal degradation [18, 27]. The mechanisms regulating the fusion proteins stability in response to TRK inhibitor remain elusive. The E3 ubiquitin ligase responsible for the degradation of the ETV6::TRKC fusion has been identified (i.e., KPC1) with 3 out of 5 lysine residues predicted to be ubiquitinated in the ETV6 portion [27]. A proteasomal degradation through ETV6 could explain the difference with the LMNA::TRKA fusion, whose stability is not affected by TRK inhibitor treatment. Moreover, studies in mouse model suggest that the ubiquitination of TrkA controls the signal transduction and occurs via the noncanonical K63 ubiquitin chains [28] responsible for regulating mechanisms like endocytosis, protein/protein interaction and protein trafficking rather than protein degradation. Overall, these data suggest that inhibition of the mechanisms stabilizing an oncogenic fusion or the stimulation of the pathways inducing its degradation may represent alternative therapeutic approaches in the context of TRK inhibitor resistant cases.

Entrectinib and Larotrectinib are type I inhibitors that occupy the ATP pocket of the kinase and demonstrate the same efficacy in cancer patient treatment. Larotrectinib is the most specific inhibitor, targeting TRKA, TRKB and TRKC, while Entrectinib, in addition to TRKs, can inhibit ROS1 and ALK. Pharmacodynamic studies showed that Entrectinib has a half-life 10 times higher than Larotrectinib (20 h vs 2 h). These characteristics could explain, for example, why our models show similar sensitivity to both drugs despite the different concentrations. In the case of apoptosis assay, Entrectinib induces the same levels of Annexin V as Larotrectinib at a concentration 10 times lower. While it is known that ERK/MAPK and PI3K/AKT inhibition control apoptosis [29], the Annexin V assay scores only one of the effects of TRK inhibition. We hypothesize that the difference between the two drugs may reside in the multi-kinase activity of Entrectinib that could deregulate other pathways leading to increase in apoptosis. Finally, another aspect that should be considered when comparing our experimental settings and the efficacy in cancer therapy is the duration of our experiment (72 h) vs the patient’s treatment that spans over a period of several weeks depending on the adopted regimen. In summary, our findings highlight the flexibility of our system in generating cancer relevant aberrations and the importance of the cellular context when generating in vitro models. Moreover, our models provide a tool to study the tumor-acquired resistance to TRK inhibitors and to identify druggable targets exploiting tumor vulnerabilities.

## Materials and methods

### Mammalian cell culture

All experiments were approved by the Tri-SCI Embryonic Stem Cell Research Oversight Committee (ESCRO). Human embryonic stem cells (WA01, H1) are available from WiCell under a material transfer agreement and were cultured in mTESR Plus medium (Stemcell technologies, #100-0276) on vitronectin-coated plates (Life technologies, #A14700). Human-derived mesenchymal progenitors (hES-MP) were maintained in MEM medium with 10% Hyclone FBS (Fisher Scientific #SH30070.03). Human colorectal carcinoma (CRC) *TPM3::NTRK1* fusion positive KM12 cells (a gift from Maurizio Scaltriti, MSKCC) were grown in RPMI with 10% FBS. Retinal Pigment Epithelium (RPE) cells were maintained in DME-HG:F-12 with 15 mM Hepes, 2.5 mM l-Glutamine, 2.4 g/L Sodium Bicarbonate with 10% FBS and Pen/Strep.

### Generation of donor plasmid for targeting and sgRNAs

For the generation of the hygromycin-based donor template, 372-bp-long homology arm from *LMNA* and 433-bp-long homology arm *NTRK1* were amplified by hES cells and cloned at the NotI-NheI (*LMNA*) and SalI-ApaI (*NTRK1*) in a plasmid previously described [8]. The frame of the plasmid was modified to allow the expression of the hygromycin gene from the *LMNA* gene promoter. For the generation of the puromycin based plasmid, the hygromycin marker was excised and replaced by the puromycin sequence at the AavrII-SalI sites. A new 461-bp-long homology arm was cloned at the NotI-NheI. The *ETV6* and *NTRK3* homology arms were amplified from hES cells and cloned into MV-PGK-Puro-TK [8] at the NotI-NheI and XhoI-AscI sites. The primers for the amplification of the homology arms are listed in Supplementary Table S2. sgRNA sequences were cloned into the dual Cas9/sgRNA expression vector pSpCas9(BB)-2A-Puro (PX459) (Addgene #48139) according to published protocol [30]. The oligos used are listed in Supplementary Table S3.

### Generation of cell lines

Wild-type hES cells were transfected by Amaxa nucleofector with 3 µg of each plasmid (sgRNAs and donor plasmid), plated and selected with 150 µg/ml hygromycin or 0.5 µg/ml puromycin for 8 days. Selected colonies were transferred in 96-well plate and screened for correct integration of donor template. For the generation of the NHEJ deletion mutant cells were transfected with sgRNAs and after a transient selection in puromycin (0.5 µg/ml) for 24 h, were plated for single-cell colonies formation. Clones were screened by genomic DNA PCR and the breakpoint junctions were analyzed by Sanger sequencing.

### PCR and RT-PCR and qRT-PCR analysis

Genomic DNA and RNA were extracted as previously described [8]. PCR and RT-PCR were performed using Thermo Scientific Dream Taq Green PCR master mix (Thermo Scientific). qRT-PCR was done as previously described [13]. Primers and PCR conditions are listed in Supplementary Table S4.

### Isolation of clones after removal of selectable marker and differentiation to mesenchymal progenitors

hES cells expressing either the hygromycin or puromycin marker were transfected with 3 µg of a plasmid expressing Cre recombinase. 48 h after transfection cells were plated for single-cell colony formation and screened by PCR for removal of selectable marker. The constitutive expression of the fusion was confirmed by RT-PCR. Expression of fusions in hES-MP cells was also obtained with a self-deleting lentivirus expressing Cre [8]. The hES cells before and after removal of selectable marker were differentiated to mesenchymal progenitor (hES-MP) using a commercially available kit (STEMCELL technologies #05240).

### Fluorescence in situ hybridization

FISH analysis was performed using custom BAC probes flanking *LMNA* and *NTRK1* as well as *ETV6* genes, as previously described. Metaphases spreads were applied on slides and were imaged using the metasystem (Zeiss Imager.2 and ISIS 5.2) (Metasystems, Boston, USA), using standard protocol [13].

### Immunohistochemistry

Immunohistochemistry staining was performed on formalin-fixed, paraffin embedded cytospin pellet for TRKA and TRKC on hES-MP cells expressing the LMNA::TRKA and ETV6::TRKC fusion proteins, respectively, using a commercially available pan-TRK monoclonal antibody, clone EPR17341 (Abcam, Cambridge, MA) on Ventana machine, following standard protocols.

### Treatment with TRK inhibitors

Cells were plated on six-well or 6-cm plate, treated for 24, 48, and 72 h at different concentrations of Entrectinib (Bio Vision #1324) and Larotrectinib (Selleckchem LOXO-101 S7960) and harvested for western blot and apoptosis assay. For viability assay cells were treated on 96-well plates for 7 days and viability assay performed using the Cell Titer Glo (Promega, G9242) following the manufacturer instructions.

### Western blot

Protein extraction and western blot analysis were performed as previously described [13]. Antibodies were prepared as follow: TRKA (CST #30697 S) 1:1000 in 5% milk, phospho TRKA/TRKB (CST #4621 S) 1:1000 in TBS (0.1% Tween, 5% BSA), phospo ERK1/2 (CST #4370 T), 1:2000 in TBS (0.1% Tween, 5% BSA), ERK1/2 (CST #9102) 1:1000 in TBS (0.1% Tween, 5% BSA), Clathrin 1:20,000 (BDBiosciences #610499) in TBS (0.1% Tween, 5% BSA),β-actin (CST #4970 S) 1:1000 in 5% milk. TRK (pan) (CST#92991) 1:1000 in 5% milk.

### Apoptosis assay

Cells were collected after treatment with TRK inhibitors and labeled using FITC Annexin V kit (Biolegend, 640905) according to manufacturer’s instruction and propidium iodide (PI), followed by flow cytometry and quantification by FlowJo software. Data are presented as the ratio of the percentage of AnnexinV/PI positive cells in untreated versus treated cells.

### Whole transcriptome sequencing

RNA sequencing (RNAseq) was performed in clones 1.14 and 13 in both hES and hES-MP background and compared to wild-type hES and hES-MP cell lines using the Illumina protocol [31]. All reads were aligned with STAR (ver 2.3) and BowTie2 against the human reference genome (hg19). Individual comparisons between each *LMNA::NTRK1* positive clone (1.14 or 13) vs wild-type in similar cellular contexts were performed and differentially expressed genes (DEG) were obtained using log2 fold change (FC) > 2.

### GSEA analysis

The gene lists and their log2FC from each comparison described above were subjected to gene set enrichment analysis (GSEA) using Bioconductor clusterProfiler package to identify significant pathways [32]. The curated gene sets and ontology gene sets [33] were used as the pathway database.

### Statistical analysis

Analysis was performed using the Graphpad software. Number of experiments is indicated in figure legends. Numerical data are shown as the mean ± s.d and differences between groups were determined using unpaired *t*-test. *p*-value < 0.05 was considered statistically significant. If not specified, the analysis is not significant.

## Supplementary information

Suppl Fig. 1

Suppl Fig. 2

Suppl Fig. 3

Supplementary Figure Legends

Supplementary Tables

## Data Availability

Data have been deposited in sequence read archive (SRA) database under accession number PRJNA922933.
