# Supplementary figures and images for: Generating in vitro models of NTRK-fusion mesenchymal neoplasia as tools for investigating kinase oncogenic activation and response to targeted therapy
Source: Oncogenesis. 2023 Feb 17;12(1):8. doi: 10.1038/s41389-023-00454-6 (PMC9938185; doi:10.1038/s41389-023-00454-6)

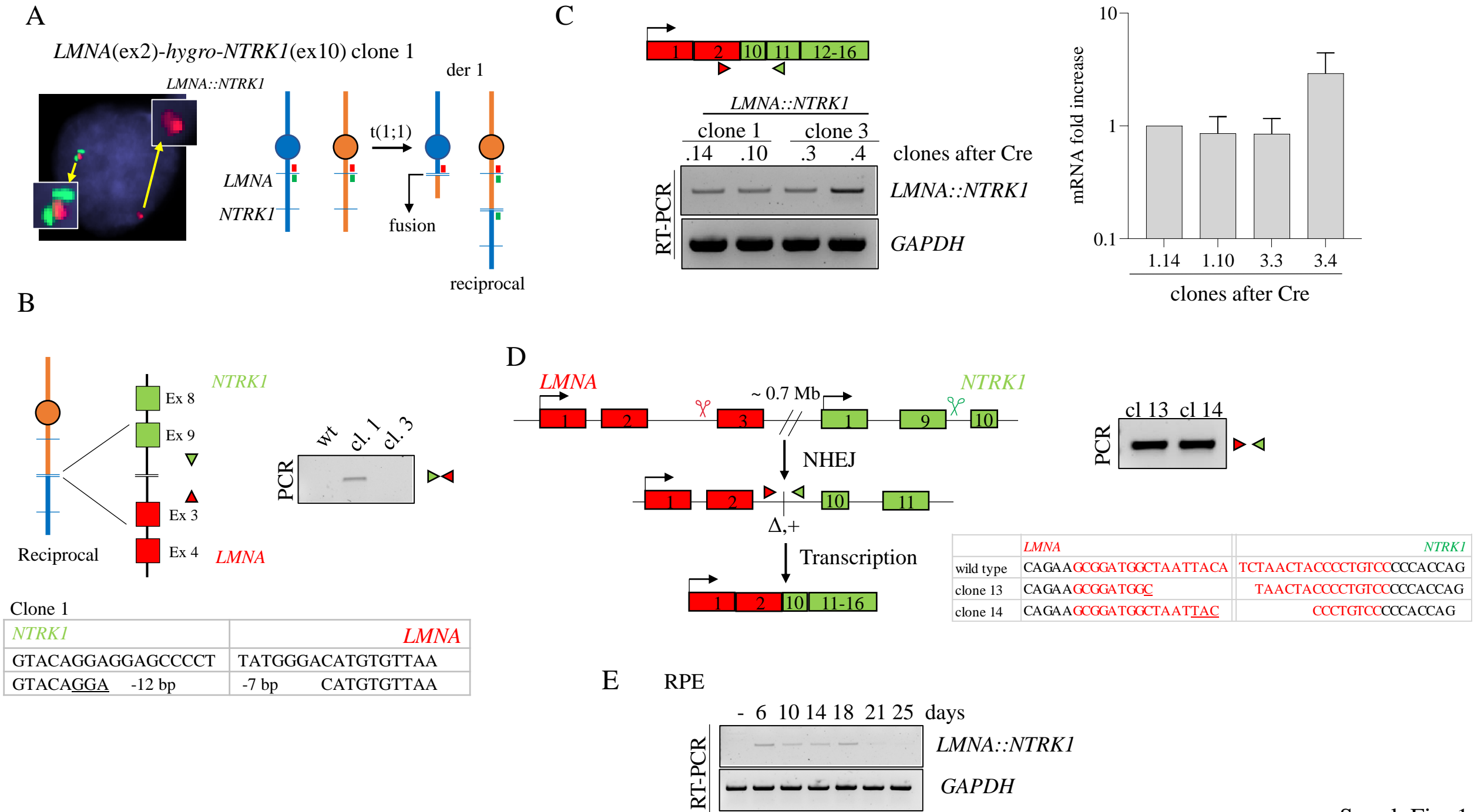

Suppl. Fig. 1

Supplement: Supplementary file 1 — Suppl Fig. 1 [file 41389_2023_454_MOESM1_ESM.pdf]

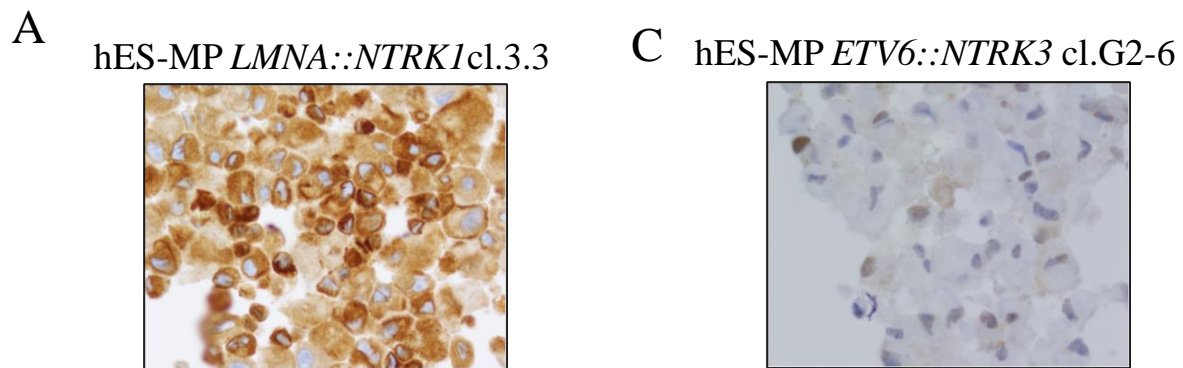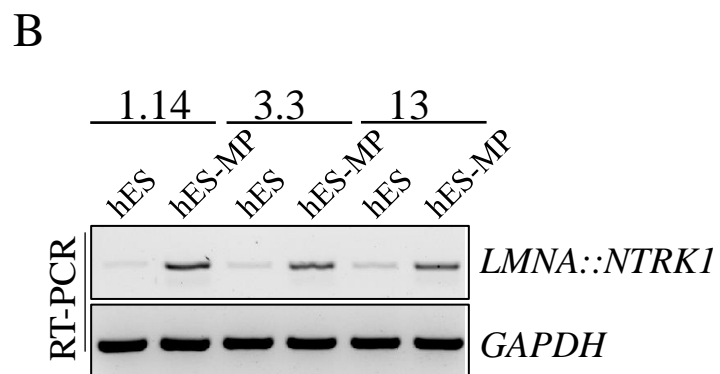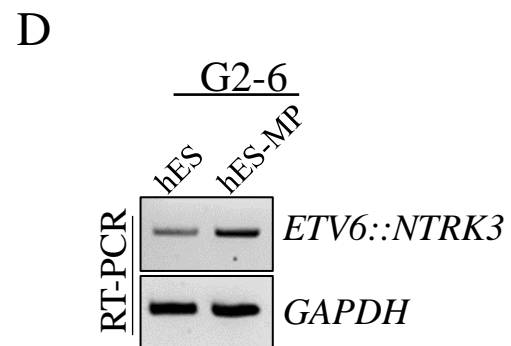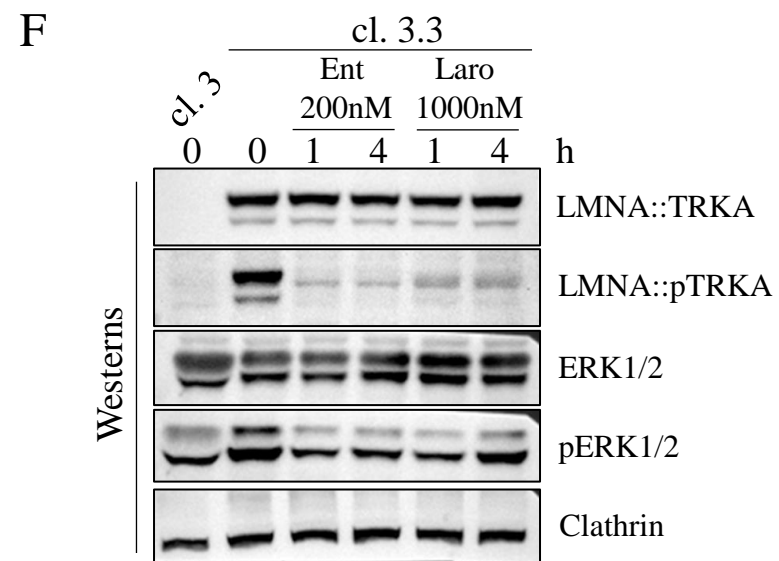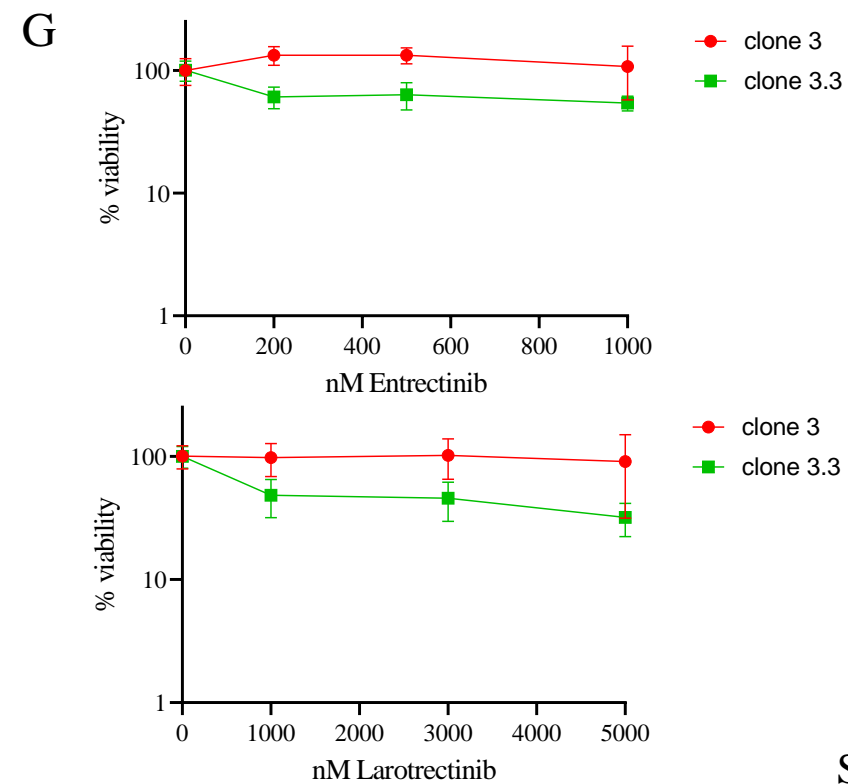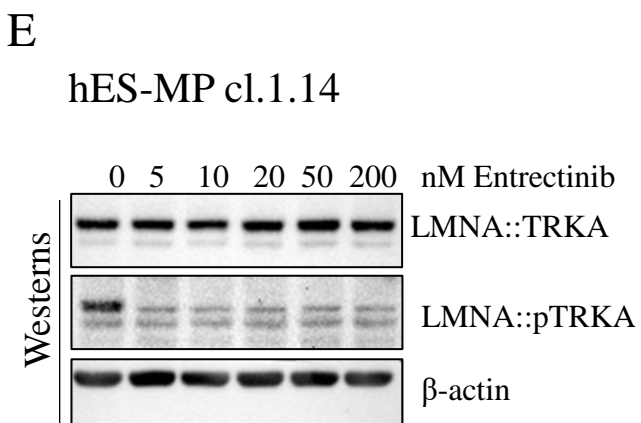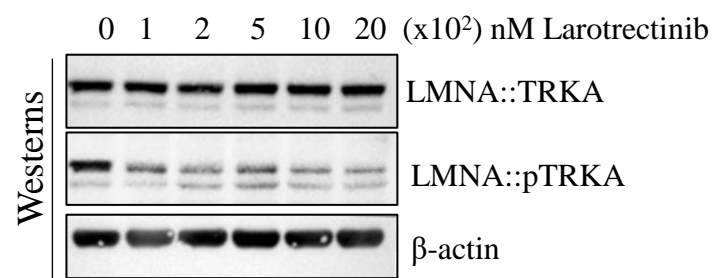

Supplement: Supplementary file 3 — Suppl Fig. 3 [file 41389_2023_454_MOESM3_ESM.pdf]
