# Supplementary material for: Generating in vitro models of NTRK-fusion mesenchymal neoplasia as tools for investigating kinase oncogenic activation and response to targeted therapy
Source: Oncogenesis. 2023 Feb 17;12(1):8. doi: 10.1038/s41389-023-00454-6 (PMC9938185; doi:10.1038/s41389-023-00454-6)
Supplement: Supplementary file 2 — Suppl Fig. 2 [file 41389_2023_454_MOESM2_ESM.pdf]

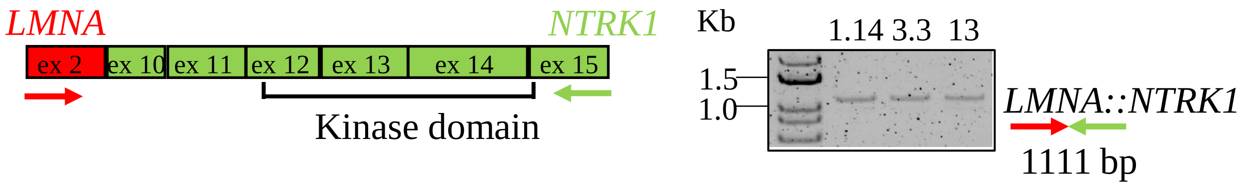

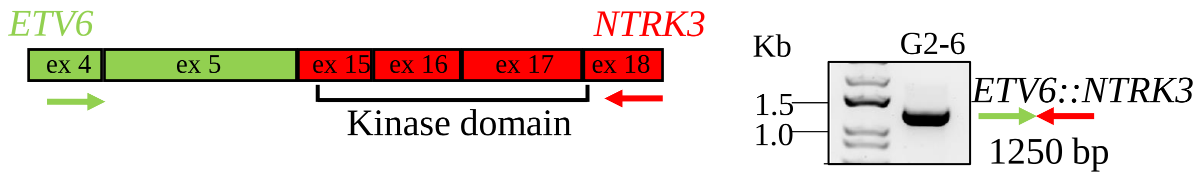

LMNA-NTRK1

CAATACCAAGAAGGAGGGTGACCTGATAGCTGCTCAGGCTCGGCTGAAGGACCTGGA
GGCTCTGCTGAACTCCAAGGAGGCCGCACTGAGCACTGCTCTCAGTGAGAAGCGCAC
GCTGGAGGGCGAGCTGCATGATCTGCGGGGCCAGGTGGCCAAG **(*LMNA* exon 2)**

GTCTCGGTGGCTGTGGGCCTGGCCGTCTTTGCCTGCCTCTTCCTTTCTACGCTGCTCCTT
GTGCTCAACAAATGTGGACGGAGAAACAAGTTTGGGATCAACC **(*NTRK1* exon 10)**

GCCCGGCTGTGCTGGCTCCAGAGGATGGGCTGGCCATGTCCCTGCATTTCATGACATT
GGGTGGCAGCTCCCTGTCCCCCACCGAGGGCAAAGGCTCTGGGCTCCAAGGCCACATC
ATCGAGAACCCACAATACTTCAGTGATGCCT **(*NTRK1* exon 11)**

GTGTTCACCACATCAAGCGCCGGGACATCGTGCTCAAGTGGGAGCTGGGGGAGGGCG
CCTTTGGGAAGGTCTTCCTTGCTGAGTGCCACAACCTCCTGCCTGAGCAGGACAAGAT
GCTGGTGGCTGTCAAG **(*NTRK1* exon 12)**

GCACTGAAGGAGGCGTCCGAGAGTGCTCGGCAGGACTTCCAACGTGAGGCTGAGCTG
CTCACCATGCTGCAGCACCAGCACATCGTGCGCTTCTTCGGCGTCTGCACCGAGGGCC
GCCCCCTGCTCATGGTCTTTGAGTATATGCGGCACGGGGACCTCAACCGCTTCCTCCG
(*NTRK1* exon 13)

ATCCCATGGACCTGATGCCAAGCTGCTGGCTGGTGGGGAGGATGTGGCTCCAGGCCCC
CTGGGTCTGGGGCAGCTGCTGGCCGTGGCTAGCCAGGTCGCTGCGGGGATGGTGTACC
TGGCGGGTCTGCATTTTGTGCACCGGGACCTGGCCACACGCAACTGTCTAGTGGGCCA
GGGACTGGTGGTCAAGATTGGTGATTTTGGCATGAGCAGGGATATCTACAGCACCGA
CTATTACCGT **(*NTRK1* exon 14)**

GTGGGAGGCCGCACCATGCTGCCCATTCGCTGGATGCCGCCCGAGAGCATCCTGTACC
GTAAGTTCACCACCGAGAGCGACGTGTGGAGCTTCGGCGTGGTGCTCTGGGAGATCTT
CACCTACGGCAAGCAGCCCTGGTACCAGCTCTCCAACACGGAG **(*NTRK1* exon 15)**

ETV6-NTRK3

GTGATGTGCTCTATGAACTCCTTCAGCATATTCTGAAGCAGAGGAAACCTCGGATTCTTTTTCA
CCATTCTTCCACCCTGGAAACTCTATACACACACAGCCGGAGGTCATACTGCATCAGAACCATG
AAGAAG **(*ETV6* exon 4)**

ATAACTGTGTCCAGAGGACCCCCAGGCCATCCGTGGATAATGTGCACCATAACCCTCCCACCAT
TGAACTGTTGCACCGCTCCAGGTCACCTATCACGACAAATCACCGGCCTTCTCCTGACCCCGAGC
AGCGGCCCCTCCGGTCCCCCCTGGACAACATGATCCGCCGCCTCTCCCCGGCTGAGAGAGCTCA
GGGACCCAGGCCGCACCAGGAGAACAACCACCAGGAGTCCTACCCTCTGTCAGTGTCTCCCATG
GAGAATAATCACTGCCCAGCGTCCTCCGAGTCCCACCCGAAGCCATCCAGCCCCCGGCAGGAGA
GCACACGCGTGATCCAGCTGATGCCCAGCCCCATCATGCACCCTCTGATCCTGAACCCCCGGCAC
TCCGTGGATTTCAAACAGTCCAGGCTCTCCGAGGACGGGCTGCATAGGGAAGGGAAGCCCATCA
ACCTCTCTCATCGGGAAGACCTGGCTTACATGAACCACATCATGGTCTCTGTCTCCCCGCCTGAA
GAGCACGCCATGCCCATTGGGAGAATAGCAG **(*ETV6* exon 5)**

ATGTGCAGCACATTAAGAGGAGAGACATCGTGCTGAAGCGAGAACTGGGTGAGGGAGCCTTTG
GAAAGGTCTTCCTGGCCGAGTGCTACAACCTCAGCCCGACCAAGGACAAGATGCTTGTGGCTGT
GAAG **(*NTRK3* exon 15)**

GCCCTGAAGGATCCCACCCTGGCTGCCCGGAAGGATTTCCAGAGGGAGGCCGAGCTGCTCACCA
ACCTGCAGCATGAGCACATTGTCAAGTTCTATGGAGTGTGCGGCGATGGGGACCCCCTCATCAT
GGTCTTTGAATACATGAAGCATGGAGACCTGAATAAGTTCCTCAG **(*NTRK3* exon 16)**

GGCCCATGGGCCAGATGCAATGATCCTTGTGGATGGACAGCCACGCCAGGCCAAGGGTGAGCTG
GGGCTCTCCCAAATGCTCCACATTGCCAGTCAGATCGCCTCGGGTATGGTGTACCTGGCCTCCCA
GCACTTTGTGCACCGAGACCTGGCCACCAGGAACTGCCTGGTTGGAGCGAATCTGCTAGTGAAG
ATTGGGGACTTCGGCATGTCCAGAGATGTCTACAGCACGGATTATTACAGG **(*NTRK 3* exon 17)**

GTGGGAGGACACACCATGCTCCCCATTCGCTGGATGCCTCCTGAAAGCATCATGTACCGGAAGT
TCACTACAGAGAGTGATGTATGGAGCTTCGGGGTGATCCTCTGGGAGATCTTCACCTATGGAAA
GCAGCCATGGTTCCAACTCTCAAACACGGAG **(*NTRK3* exon 18)**

Suppl. Fig. 2
