# Supplementary material for: Generating in vitro models of NTRK-fusion mesenchymal neoplasia as tools for investigating kinase oncogenic activation and response to targeted therapy
Source: Oncogenesis. 2023 Feb 17;12(1):8. doi: 10.1038/s41389-023-00454-6 (PMC9938185; doi:10.1038/s41389-023-00454-6)
Supplement: Supplementary file 4 — Supplementary Figure Legends [file 41389_2023_454_MOESM4_ESM.pdf]

Supplementary Figure Legends

Supplementary Figure 1. Analysis of clones expressing the *LMNA::NTRK1* oncogenic fusion. (A) Dual color FISH analysis of clone 1 (*LMNA::NTRK1*) showing the *LMNA* locus (red, centromeric; green, telomeric), confirming the t(1;1) pattern. **(B)** Schematic representation of the t(1;1) reciprocal translocation and PCR on clones 1 and 3 for amplification of breakpoint junction with sequence from clone 1. **(C)** RT-PCR and qRT-PCR comparison of the *LMNA::NTRK1* fusion transcript level in sub-clones isolated from clone 1 (1.14 and 1.7) and clone 3 (3.3 and 3.4) after removal of the selectable marker by Cre recombinase expression. **(D)** Strategy for the generation of *LMNA::NTRK1* deletion mutant by NHEJ. Two DSBs are induced at the *LMNA* intron 2-3 and *NTRK1* intron 9-10 and clones are screened for positive PCR across the breakpoint junction. Sequences of the junction in clones 13 and 14 show accumulation of deletions near the DSB sites. Sequences in red represent the sgRNA binding site. **(E)** Time course (days) experiment for *LMNA::NTRK1* fusion detection in RPE cells after expression of *LMNA* and *NTRK1* sgRNAs.

Supplementary Figure 2. Sequences of the *LMNA::NTRK1* and *ETV6::NTRK3* transcripts expressed in hES-MP isogenic cell lines. Left panel, Diagram with primers position for the amplification of the *LMNA::NTRK1* fusion and Sanger sequence of RT-PCR product. Nucleotides highlighted in turquoise on *NTRK1* exons 12 and 15 represent the codons of the first (Histidine, H) and last (Valine, V) amino acids of kinase domain. The codons highlighted in yellow on exon 13 carries a polymorphism (G>A) in the third position (in green) not altering the amino acid sequence. Nucleotides in grey code for residues tyrosine-Y 680/681 autophosporylated after TRKA activation. Underlined are the sequences of primers used for RT-PCR amplification and Sanger sequence. Right panel, Diagram with the primers for the amplification of the *ETV6::NTRK3* fusion in clone G2-6 and sequence of the transcript. Nucleotide in turquoise (exons 15 and 18) mark the beginning and the end of the kinase domain. Nucleotides in grey code for the tyrosine residues 709/710 phosporylated after activation of the kinase.

Supplementary Figure 3. Fusion localization, expression and TRKA, ERK1/2 phosphorylation after Entrectinib and Larotrectinib treatment. (A) TRKA expression in hES-MP cells expressing the LMNA::TRKA fusion protein (clone 3.3) with cytoplasmatic and perinuclear localization (pan-TRK ICC, 40x magnification). **(B)** RT-PCR expression of *LMNA::NTRK1* in hES cells and hES-MP showing difference in the transcript levels. **(C)** ETV6::TRKC expression and nuclear localization in hES-MP cells expressing the *ETV6::NTRK3* fusion (clone G2-6) (pan-TRK ICC, 40x magnification). **(D)** RT-PCR comparing *ETV6::NTRK3* expression in hES vs hES-MP **(E)** Western blot analysis for TRKA phosphorylation and LMNA::TRKA fusion protein on clone 1.14 at increasing concentration of Entrectinib (0, 5, 10, 20, 50, 200 nM) and Larotrectinib (0, 100, 200, 500, 1000 and 2000 nM). **(F)** Western blot for

ERK1/2 phosphorylation in cells expressing the *LMNA::NTRK1* fusion after 1 and 4 hours exposure to 200 nM Entrectinib and 1000 nM Larotrectinib. **(G)** Viability assay on cells expressing the *LMNA::NTRK1* fusion (clones 3.3) after continuous exposure (7 days) to increasing concentration of Entrectinib and Larotrectinib. Results are presented as the mean of 3 independent experiments.
