# Supplementary material for: Generating in vitro models of NTRK-fusion mesenchymal neoplasia as tools for investigating kinase oncogenic activation and response to targeted therapy
Source: Oncogenesis. 2023 Feb 17;12(1):8. doi: 10.1038/s41389-023-00454-6 (PMC9938185; doi:10.1038/s41389-023-00454-6)
Supplement: Supplementary file 5 — Supplementary Tables [file 41389_2023_454_MOESM5_ESM.docx]

**Generating *in vitro* models of NTRK-fusion mesenchymal neoplasia as tools for investigating kinase oncogenic activation and response to targeted therapy**

**Supplementary Table S1. GSEA enrichment analysis results.**

**Ferreira_Ewings_Sarcoma_Unstable_vs_Stable_Up**

| **ID** | **Set Size** | **Enrichment Score** | **NES** | **P value** | **P adjust** | **FDR** | **Rank** | **Leading_edge** |
| --- | --- | --- | --- | --- | --- | --- | --- | --- |
| Clone 13 hES-MP | 145 | 0.853 | 2.554 | 1e-10 | 6.08e-10 | 9.46e-11 | 3006 | tags=63%, list=11%, signal=56% |
| Clone 1.14 hES-MP | 145 | 0.828 | 2.516 | 1e-10 | 5.77e-10 | 9.05e-11 | 1914 | tags=50%, list=7%, signal=47% |
| Clone 13 hES | 145 | 0.693 | 2.155 | 1e-10 | 7.30e-10 | 1.42e-10 | 6160 | tags=40%, list=23%, signal=31% |

**Nakayama_Soft_Tissue_Tumors_PCA2_Up**

| **ID** | **Set Size** | **Enrichment Score** | **NES** | **P value** | **P adjust** | **FDR** | **Rank** | **Leading_edge** |
| --- | --- | --- | --- | --- | --- | --- | --- | --- |
| Clone 13 hES-MP | 81 | 0.892 | 2.499 | 1e-10 | 6.08e-10 | 9.46e-11 | 627 | tags=46%, list=2%, signal=45% |
| Clone 1.14 hES-MP | 81 | 0.898 | 2.560 | 1e-10 | 5.77e-10 | 9.05e-11 | 691 | tags=49%, list=3%, signal=48% |
| Clone 13 hES | 81 | 0.676 | 1.959 | 5.44e-6 | 1.85e-5 | 3.59e-6 | 4414 | tags=30%, list=16%, signal=25% |

**Supplementary Table S2. List of primers for cloning of homology arms (the cloning sites are underlined).**

| **Gene** | **Forward Primer** | **Reverse Primer** |
| --- | --- | --- |
| *LMNA*-HA1 | 5’- AAAAAGCGGCCGCATCTCTAGAAAATAAAAATT -3’ | 5’- AAAAAGCTAGCGCCATCCGCTTCTGATGGCC -3’ |
| *LMNA*-HA2 | 5’- AAAAGCGGCCGCGCCTGGGCAACAGAGTGAGA -3’ | 5’- AAAAGCTAGCCCCTTGTATACCGGAGGGCC -3’ |
| *NTRK1-*HA | 5’- AAAAAGTCGACCCCACCAGGTCTCGGTGGCT -3’ | 5’- AAAAAGGGCCCCTGGCCCCATGGGCACCAAA -3’ |
| *ETV6*-HA | 5’- AAAAGCGGCCGCTCAACCTCTCTCATCGGGAA -3’ | 5’- AAAAGCTAGCGAATTACAGGCCTGAATCTG -3’ |
| *NTRK3*-HA | 5’- AAAAGTCGACGAGGGGCAGATGGGCAAGGG -3’ | 5’- AAAAGGGCCCACTGAGTCACATAGAGAAGT-3’ |

**Supplementary Table S3. List of oligos for preparation of gRNAs (underlined sequences represent the DNA bound by gRNA).**

| Gene | Position | sense | antisense |
| --- | --- | --- | --- |
| gRNA*^LMNA^*^-1^ | intron 2-3 | 5’- CACCGGCGGATGGCTAATTACATAT -3’ | 5’- AAACATATGTAATTAGCCATCCGCC -3’ |
| gRNA*^LMNA^*^-2^ | intron 2-3 | 5'- CACCGAAATCACAAGGGCCTATAGG -3' | 5'- AAACCCTATAGGCCCTTGTGATTTC -3' |
| gRNA*^NTRK1^*^-10^ | intron 9-10 | 5'- CACCGGGACAGGGGTAGTTAGATCC -3’ | 5'-AAACATACGCAGCACAACTTATCCC -3' |
| gRNA*^ETV6^*^-5^ | intron 5-6 | 5'- CACCGTGGCGTTACCGCTCACAGGA -3' | 5'- AAACTCCTGTGAGCGGTAACGCCAC -3' |
| gRNA*^NTRK3^*^-15^ | intron 14-15 | 5’- GAGGGGCAGATGGGCAAGGG -3’ | 5’- AAACCCGTGCGGGTTATCGACCTCC -3’ |

**Supplementary Table S4. List of primers for RT-PCR and PCR analysis.**

| **Product** | **Sequence** |
| --- | --- |
| *LMNA::NTRK1* Fusion  RT-PCR  T_a_=61°, 30 sec. | Forward 5’-CAATACCAAGAAGGAGGGTG-3’ |
|  | Reverse 5’-GTGGGTTCTCGATGATGTGG-3’ |
| *ETV6::NTRK3* Fusion  RT-PCR  T_a_=61°, 30 sec. | Forward 5’- GCATCAGAACCATGAAGAAG-3’ |
|  | Reverse 5'- CCTCTTAATGTGCTGCACAT-3' |
| *LMNA::NTRK1* Fusion  RT-PCR  T_a_=58°, 1 m. | Forward 5’-CAATACCAAGAAGGAGGGTG-3’ |
|  | Reverse 5’-CTCCGTGTTGGAGAGCTGG-3’ |
| *ETV6::NTRK3* Fusion  RT-PCR  T_a_=58°, 1 m 30 sec. | Forward 5’- GCATCAGAACCATGAAGAAG-3’ |
|  | Reverse 5'- ATGGCTGCTTTCCATAGGTG-3' |
| Reciprocal Translocations PCR  T_a_=60°, 30 sec. | Forward 5’-TAGAAGCTTGTGCAGACTTT-3’ |
|  | Reverse 5’-GCCTCACAAGTAGTTGTTGGG-3’ |
| Breakpoint Junction  PCR  T_a_=60°, 30 sec. | Forward 5’- CTCCCAGTTTGGCCCTCCG -3’ |
|  | Reverse 5'- GATCCCAAACTTGTTTCTCC -3' |
